# Perinatal outcomes of frequent attendance in midwifery care in the Netherlands: a retrospective cohort study

**DOI:** 10.1186/s12884-020-02957-1

**Published:** 2020-05-06

**Authors:** Janneke T. Gitsels-van der Wal, Lisanne A. Gitsels, Angelo Hooker, Paula Scholing, Linda Martin, Esther I. Feijen-de Jong

**Affiliations:** 1 Amsterdam UMC, Vrije Universiteit Amsterdam, Midwifery Science, AVAG, Amsterdam Public Health research institute, Van der Boechorststraat 7, 1081 HV Amsterdam, The Netherlands; 2 ESRC funded Business and Local Government Data Research Centre (BLG DRC), School of Computing Sciences, University of East Anglia, Norwich Research Park, Norwich, NR4 7TJ UK; 3 University College London, London, UK; 4 Department of Obstetrics and Gynaecology, Amsterdam UMC, Vrije Universiteit Amsterdam, De Boelelaan, 1117 Amsterdam, Netherlands; 5 Zaans Medical Center, Department of Obstetrics and Gynecology, Koningin Julianaplein 58, Zaandam, Netherlands; 6 Department of General Practice & Elderly Medicine, University of Groningen, University Medical Center Groningen, POBox 30001, 9700 RB Groningen, the Netherlands

**Keywords:** Midwifery, Obstetric delivery, Perinatal outcomes, population health, Frequent attendance, Pain relief, Induced labour, Healthcare utilisation, Patient acceptance of health care, Low-risk women

## Abstract

**Background:**

Over the last decade, a trend towards high utilisation of primary maternity care was observed in high-income countries. There is limited research with contradictory results regarding frequent attendance (FA) and perinatal outcomes in midwifery care. Therefore, this study examined possible associations between FA in midwifery care and obstetric interventions and perinatal outcomes.

**Methods:**

A retrospective cohort study was performed in a medium-sized midwifery-led care practice in an urban region in the Netherlands. Frequent attenders (FAs) were categorised using the Kotelchuck-Index Revised. Regression analyses were executed to examine the relationship between FAs and perinatal outcomes, stratified by antenatal referral to an obstetrician. Main outcomes of interest were Apgar score ≤ 7 and perinatal death, birth weight, mode of delivery, haemorrhage, place of birth, transfer during labour, and a requirement for pain relief.

**Results:**

The study included 1015 women, 239 (24%) FAs and 776 (76%) non-FAs, 538 (53%) were not referred and 447 (47%) were referred to an obstetrician. In the non-referred group, FA was significantly associated with a requirement for pain relief (OR 1.98, 95% CI 1.24–3.17) and duration of dilatation (OR 1.20, 95% CI 1.04–1.38). In the referred group, FA was significantly associated with induction of labour (OR 1.86, 95% CI 1.17–2.95), ruptured perineum (OR 0.50, 95% CI 0.27–0.95) and episiotomy (OR 0.48, 95% CI 0.24–0.95). In the non-referred and the referred group, FA was not associated with the other obstetric and neonatal outcomes. Due to small numbers, we could not measure possible associations of FA with an Apgar score ≤ 7 and perinatal death.

**Conclusion:**

In our study, perinatal outcomes differed by FA and antenatal referral to an obstetrician. In the non-referred group, FA was significantly associated with medical pain relief and duration of dilatation. In the referred group, FA was significantly associated with induction of labour, ruptured perineum, and episiotomy. Further research with a larger study population is needed to look for a possible association between FA and primary adverse birth outcomes such as perinatal mortality.

## Background

Frequent attendance (FA) has become a growing health problem and a central issue in primary health care systems [1–3]. FA leads to a higher workload and pressure on the healthcare system and does not improve health outcomes per se. FA is defined as the top 10% of the total number of visits during 1 year in practice, and its prevalence has increased from 2 to 20% in the last two decades [2, 4–7]. Factors contributing to higher rates of FA are age, educational level, ethnicity, living area, body mass index (BMI), smoking and alcohol consumption [1, 2, 8–15]. Despite a five-fold increase in costly hospital referrals, frequent attenders (FAs) showed repeatedly multi-morbid health disorders such as psychiatric illness, emotional distress, social difficulties and reduced self-reported quality of life [1, 2, 5, 16–18]. The trend towards increased FA has also been observed in maternity health care [19–23]. Guidelines about the appropriate number of antenatal visits among low-risk women differ worldwide, due to diverse prenatal health care systems, ranging between eight to 14 visits [20, 24–26]. During the last decade, FA in the Netherlands increased from 13 to 23% [23]. Previous studies showed that FA in midwifery care is mainly associated with worries and vague complaints; single marital status, assisted conception, sexual violence and psychosocial problems are considered important underlying factors, which increase anxious feelings and lead to more prenatal visits [23, 27–32].

The recommended number of prenatal visits aims to establish the best possible perinatal outcomes [25]. Inadequate prenatal care, defined as a significantly lower number of visits than recommended, is associated with adverse maternal and perinatal outcomes [19, 33, 34]. However, little research has been conducted regarding the relationship between FA, obstetric interventions and perinatal outcomes in midwifery care. An American study reported an increased number of obstetric interventions (induction of labour and caesarean section) among FAs with a low-risk status for obstetric complications compared to similar low-risk non-frequent attenders (non-FAs) [27]. However, no significant differences were reported in Apgar scores or small for gestational age [27]. In contrast, a Dutch study reported no associations between FA and obstetric interventions but reported an increased risk for preterm birth, low Apgar scores, low birth weight and perinatal mortality among FA low-risk women [35]. Few studies examined the relationship between FA and perinatal outcomes among high-risk women in obstetric-led care. A prospective cohort study among women with gestational diabetes and type 2 diabetes examined a possible association between the number of prenatal visits and perinatal outcomes; results showed improved neonatal outcomes of FAs [36].

In a previous study, we assessed the prevalence of FA in Dutch midwifery care and its underlying reasons [23]. Drawing on this work, the current study aimed to examine possible associations between FA in Dutch midwifery care and perinatal outcomes (e.g. obstetric interventions and neonatal outcomes). As antenatal referral to an obstetrician was found to be an effect modifier of prenatal healthcare utilisation in previous studies [19, 23], we compared the outcomes of women with FA to those with the recommended number of visits and stratified by referral.

## Methods

### Study design

The current retrospective cohort study was performed in a midwifery practice with an average of 750 low-risk pregnant women per year and situated in an urban region close to Amsterdam, the Netherlands. Women were recruited from January 2015 to January 2017. The Medical Ethics Committee of the Amsterdam University Medical Center approved the study (ref. 2018.019).

### Participants

All women registered for prenatal care at this midwifery practice approved the use of their anonymous data for research to improve the quality of prenatal care and were eligible for inclusion. Women were excluded if they had a miscarriage, ectopic pregnancy or were referred to an obstetrician in the first trimester. Women who only received postnatal care or who were referred to another practice before giving birth were also excluded. The remaining cohort was included and grouped based on the level of health care utilisation using the Kotelchuck Index-Revised (KI-R).

The KI-R is a validated index based on the guidelines by Royal Dutch Organisation of Midwives and calculated by the number of face-to-face prenatal visits with a midwife, gestation and onset of care (Additional file 1: Appendix 1) [19, 37]. Women were categorized according to health care utilisation; inadequate (KI-R of < 50%), intermediate (50–79%), adequate (KI-R of 80–109%), and adequate plus (KI-R of > = 110%). Women with inadequate or intermediate use of care were excluded in the current analysis. The final cohort consisted of women with adequate utilisation of care (KI-R of 80–109%), classified as non-FAs and women with adequate plus the utilisation of care (KI-R of > = 110%), classified as FAs.

### Dutch maternity care context

In the Dutch maternity care system, low-risk women are guided in midwifery care in the community, and high-risk women are guided in obstetric care at the hospital; midwives refer low-risk women to obstetricians in case of (suspected) complications. Guided by midwives, low-risk women have the option to give birth at home or in a birth centre (outside or in a hospital). Induction of labour is only possible in obstetric-led care.

### Data collection and perinatal outcomes

Anonymised data were obtained from the digital maternity database [23]. These standardised data included level of health care utilisation, perinatal and neonatal outcomes, and sociodemographic and medical characteristics. Outcomes of primary concern to health professionals are Apgar Score (< 8 after 5 min of delivery) and perinatal death. Other outcomes of interest in this study were birth weight (<10th percentile for gestational age), mode of delivery (spontaneous, vaginal assisted, Caesarean section), perineum status (non-ruptured, ruptured, or episiotomy), haemorrhage (< 1000 ml), place of birth (home, birth centre or hospital), gestational age at onset of labour (in weeks), transfer during labour (e.g. because of insufficient progress of dilatation, request for pain relief or signs of fetal distress), pain relief (morphine or epidural anaesthesia), duration of dilatation (in hours), and duration of expulsion (in minutes).

The sociodemographic and medical information included: age (in years), education (low: primary school and uncompleted vocational training / intermediate: secondary school and completed vocational training / high: college or university) [23], ethnicity (Dutch/western non-Dutch/non-western non-Dutch), marital status (partner yes/no), occupation (yes/no), deprivation based on postal code (no/yes) [38], parity (nulliparous/multiparous), mode of conception (unassisted/assisted), psychosocial problems (no/yes in the past/yes at present), sexual violence (no/yes), smoking (no/yes), alcohol consumption (no/yes), drugs addiction (no/yes), body mass index (BMI, normal weight/overweight/obese) [39], and antenatal referral to obstetrician (no/yes). Psychosocial problems were defined as ‘*the broad spectrum of all complaints which are not strictly medical or somatic and affect the patient’s functioning in daily life’; for example, stress, sleep disorder, relationship problems, financial problems, housing problems and adjustment problems* [40]*.*

### Analyses

Descriptive statistics were obtained to summarise characteristics of the study population by FA and referral. For continuous variables, if normally distributed, the means and standard deviations were obtained; otherwise, the median and interquartile range were obtained. For categorical variables, frequencies and prevalences were obtained.

Regression analyses were executed to estimate the associations of FA with perinatal outcomes in non-referred and referred participants. Logistic models were conducted for binary outcomes, nominal logit models were conducted for categorical outcomes, and linear models were conducted for continuous outcomes. No models were conducted for induction of labour among non-referred as this would only happen in obstetric led care, for the same reason no models were conducted for the place of birth and transfer during labour among the referred group as this would only happen in midwifery care.

Binary variables were excluded from the regression analyses if a category was less than 5% prevalent. Categorical variables were regrouped if a category was less than 5% prevalent. Age was centred around the mean to have a meaningful baseline. The final adjusted models included the exposure and all supporting variables that were significant (*p* < .05) using backward elimination. The models’ assumptions and performances were assessed. In case variables were not normally distributed, they were log-transformed to comply with the linear model assumptions; the model’s coefficients were exponentiated, resulting in odds ratios. All analyses were performed by two researchers independently from each other in SPSS (version 24).

## Results

**Fig. 1:**
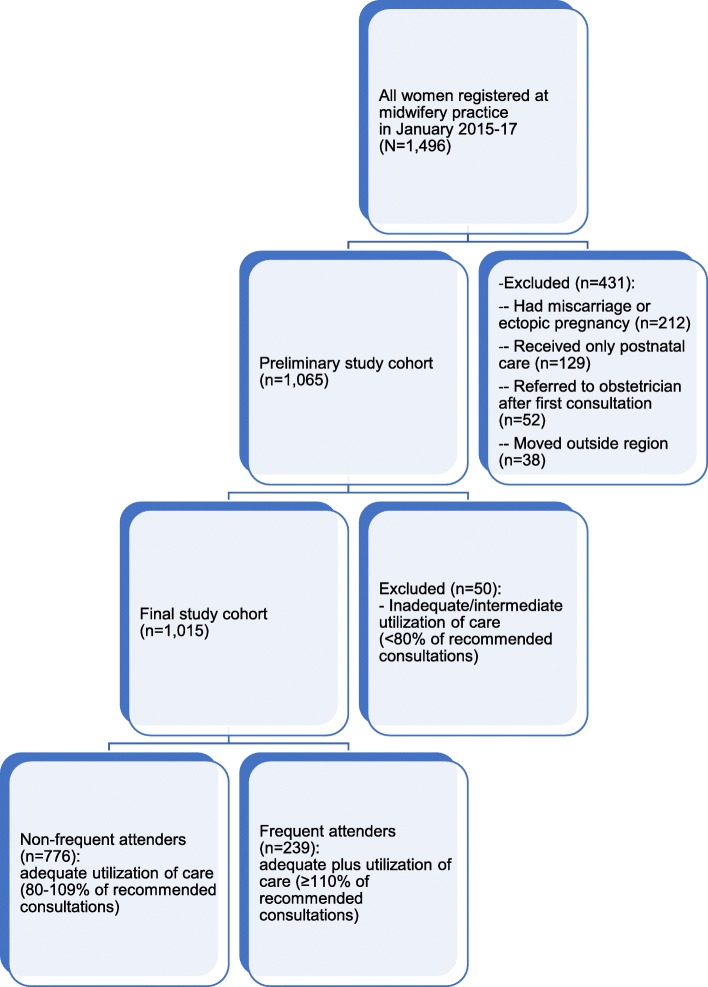
Flowchart of the eligible study population

A total of 1015 women were included in the current analysis (Fig. 1). Table 1 shows the characteristics of the study population. On average, women were aged 29 years at conception. Roughly half of the women were multiparous (53%). Approximately 48% of the women were Dutch, 8% were Western non-Dutch, and 44% were non-Western non-Dutch. One in five lived in a deprived area. Based on the utilisation of prenatal care, 239 (24%) were FAs (KI-R > 109%) and 776 (76%) were non-FAs (KI-R of 80–109%). Of the women included, 528 (53%) were not referred to an obstetrician during pregnancy, and 477 (47%) were referred (Table 2). The main reasons for referral were glucose intolerance (28%), pregnancy-induced hypertension (15%), and a previous C-section (10%) (Table 3).

**Table 1:** Characteristics of frequent attenders and non-frequent attenders (*N* = 1015)

	Complete study population N = 1015	Frequent attender *N* = 239 (23.5%)	Non-frequent attender *N* = 776 (76.5%)	***P*** value
**Consultations**				
Face-to-face Mean (sd)	11 (4.2)	14 (4.1)	10 (3.7)	<.01
**Age in years** mean (sd)	29.3 (4.9)	28.8 (5.1)	29.4 (4.8)	.24
Level of Education*				.77
Low	119 (12.1)	26 (11.3)	93 (12.4)	
Medium	459 (46.7)	112 (48.7)	347 (46.1)	
High	405 (41.2)	92 (40)	313 (41.6)	
Ethnic Background*				.08
Dutch	483 (47.6)	128 (53.6)	355 (45.8)	
Western Non-Dutch	82 (8.1)	14 (5.9)	68 (8.8)	
Non-Western Non-Dutch	449 (44.3)	97 (40.6)	352 (45.4)	
**Married/partner**	990 (97.5)	230 (96.2)	760 (97.9)	.14
Employed*	696 (69.1)	165 (69.3)	531 (69.1)	
**Deprived area**	210 (20.7)	42 (17.6)	168 (21.6)	.17
**Parity**				.24
Nulliparous	480 (47.3)	121 (50.6)	359 (46.3)	
Multiparous	535 (52.7)	118 (49.4)	417 (53.7	
Start of conception*				.03
Spontaneous	953 (94.7)	218 (92)	735 (95.6)	
Assisted	53 (5.3)	19 (8)	34 (4.4)	
Psychosocial problems*				<.01
Yes in the past	202 (20)	58 (24.3))	144 (18.6)	
Yes at present	87 (8.6)	35 (14.6)	52 (6.7)	
Sexual violence*	127 (12.5)	49 (20.6)	78 (10.1)	<.01
Smoking*	235 (23.2)	76 (31.8)	159 (20.5)	<.01
Alcohol use*	42 (4.1)	18 (7.5)	24 (3.1)	<.01
Drugs addiction*	22 (2.2)	9 (3.8)	13 (1.7)	.05
BMI*				.17
Overweight	248 (24.5)	69 (28.9)	179 (23.2)	
Obese	133 (13.2)	27 (11.3)	106 (13.7)	

*sample size varies due to missing data; valid percentages are shown

**standard deviation

**Table 2:** Characteristics of non-frequent and frequent attenders divided by referrals (N = 1015)

	Non-referred frequent attender *N* = 107 (10.5%)	Non-referred non-frequent attender *N* = 431 (42.5%)		Referred frequent attender *N* = 132 (13.0%)	Referred non-frequent attender *N* = 345 (34.0%)	
**Consultations**			<.01			<.01
Face-to-face Mean (sd)	15.8 (3.3)	12.0 (2.5)		12.6 (4.1)	7.7 (3.6)	
**Age in years**			.26			.12
mean (sd)	28.2 (5.0)	28.8 (4.7)		29.4 (5.1)	30.2 (4.9)	
Level of Education*			.56			.81
Low	8 (7.7)	48 (11.5)		18 (14.3)	45 (13.4)	
Medium	54 (51.9)	194 (46.4)		58 (46.0)	153 (45.7)	
High	42 (40.4)	176 (42.1)		50 (39.7)	137 (40.9)	
Ethnic Background*			.69			.03
Dutch	54 (50.5)	209 (48.5)		74 (56.1)	146 (42.4)	
Western Non-Dutch	9 (8.4)	48 (11.1)		5 (3.8)	20 (5.8)	
Non-Western Non-Dutch	44 (41.1)	174 (40.4)		53 (40.2)	178 (51.7)	
**Married/partner**	103 (96.3)	419 (97.2)	.62	127 (96.2)	341 (98.8)	.06
Employed*	74 (69.8)	294 (68.7)	.80	91 (68.9)	237 (69.5)	.90
**Deprived area**	19 (17.8)	76 (17.6)	.98	23 (17.4)	92 (26.7)	.04
**Parity**			.46			.29
Nulliparous	56 (52.3)	206 (47.8)		65 (49.2)	153 (44.3)	
Multiparous	51 (47.7)	225 (52.2)		67 (50.8)	192 (55.7)	
Start of conception*			.99			.16
Spontaneous	102 (96.2)	410 (96.2)		116 (88.5)	325 (94.8)	
Assisted	4 (3.8)	16 (3.8)		15 (11.5)	18 (5.2)	
Psychosocial problems*			.51			<.01
Yes in the past	25 (23.4)	80 (18.7)		33 (25.0)	64 (18.6)	
Yes at present	8 (7.5)	27 (6.3)		27 (20.5)	25 (7.2)	
Sexual violence*	17 (16.0)	37 (8.6)	.03	32 (24.2)	41 (11.9)	<.01
Smoking*	32 (29.9)	82 (19.1)	.01	44 (33.3)	77 (22.4)	.02
Alcohol use*	8 (7.5)	14 (3.3)	.05	10 (7.6)	10 (2.9)	.02
Drugs addiction*	1 (0.9)	8 (1.9)	.51	8 (6.1)	5 (1.5)	.01
BMI*			.67			.21
Overweight	23 (21.5)	91 (21.3)		46 (34.8)	88 (25.6)	
Obese	5 (4.7)	39 (9.1)		22 (16.7)	67 (19.5)	

*sample size varies due to missing data; valid percentages are shown

**standard deviation; ^#^*p* ≤ .05*;*^*~*^*p* ≤ .01

**Table 3:** Reasons for antenatal referral among the referred population (N = 477)

Reasons	N (%)
Glucose intolerance	132 (27.7)
Pregnancy-induced hypertension	70 (14.7)
Previous C-section	47 (9.9)
**Post terme** (≥42 weeks)/request for induction	30 (6.3)
Breech or other malpresentation	27 (5.7)
**Decreased** fetal movements	22 (4.6)
Intercurrent disease	17 (3.6)
Small for gestational age	16 (3.4)
Premature birth	15 (3.1)
Congenital anomaly	10 (2.1)
Large for gestational age	11 (2.3)
Anaemia	11 (2.3)
Previous small for gestational age	9 (1.9)
Hydramnion	6 (1.3)
Placenta problems	5 (1)
Uterus anomaly/cyst/myomas	5 (1)
Stillbirth	4 (.8)
Hyperemesis	2 (.4)
Fetal distress	2 (.4)
Blood loss	2 (.4)
Twin pregnancy	1 (.2)
Other^#^, such as cholestasis, maternal heart defects, rheumatism, gastric bypass, pelvic pain	33 (6.9)

^*^valid percentages are shown

### FAs versus non-FAs

FA was associated with (former) psychosocial problems (15% FA versus 7% non-FA), a history of sexual violence (21% versus 10%), an assisted start of conception (8% versus 4%), smoking (32% versus 21%) and alcohol consumption (8% versus 3%) (Table 1).

Regarding perinatal outcomes (Table 4), FA was associated with place of birth; FAs had more hospital deliveries than non-FAs (80% FA versus 72% non-FA) and less home and birth centre deliveries (3 and 17% versus 7 and 21%, respectively). Furthermore, FA was associated with induction of labour (42% versus 27% non-FA) and with medical pain relief (52% versus 38% non-FA). We found no significant differences in other outcomes.

**Table 4:** Perinatal outcomes of frequent attenders and non-frequent (N = 1015)

	Complete study population N = 1015 (100%)	Frequent attender *N* = 219 (23.5%)	Non-frequent attender N = 776 (76.5%)	***P*** value
Apgar Score after 5 min*				.93
< 8	29 (2.9)	7 (3)	22 (2.8)	
**Perinatal death**	11 (1.1)	1 (.4)	10 (1.3)	.26
**Birthweight** (in percentiles)*				.24
* P* < 10	101 (10.1)	19 (8.1)	82 (10.7)	
Mode of delivery *				.24
Spontaneous	764 (75.5)	174 (73.1)	590 (76.2)	
Vaginal assisted birth	103 (10.2)	22 (9.2)	81 (10.5)	
Caesarean section	145 (14.3)	42 (17.6)	103 (13.3)	
Perineum*				.28
Not ruptured	442 (50.7)	109 (54.8)	333 (49.5)	
Ruptured	248 (28.4)	48 (24.1)	200 (29.7)	
Episiotomy	182 (20.9)	42 (21.1)	140 (20.8)	
Haemorrhage*	69 (6.9)	12 (5.1)	57 (7.5)	.21
**Place of birth**				.01
Home	58 (5.7)	6 (2.5)	52 (6.7)	
Birth centre	215 (22.2)	41 (17.2)	163 (21)	
Hospital	742 (73.1)	192 (80.3)	561 (72.3)	
Transfer during labour*	275 (28.6)	60 (26.4)	215 (29.3)	.41
Induction of labour*	313 (30.9)	100 (42)	213 (27.4)	.00
Medical pain relief*	415 (41)	124 (52.1)	291 (37.6)	.00
**Gestational age at onset of labour in weeks** mean (sd)	39 (2.4)	38.7 (2.3)	39 (2.5)	.22
**Duration of dilatation in hours** median (IQR)	6.0 (.8)	6.0 (.9)	6.0 (.8)	.09
**Duration of expulsion in minutes** median (IQR)	16.0 (1.6)	14.9 (1.7)	16.0 (1.6)	.81

*sample size varies due to missing data; valid percentages are shown

**SD = standard deviation; IQR = interquartile range

### Non-referred pregnant women

In the non-referred group (*N* = 538), FA was associated with a history of sexual violence (16% FA versus 9% non-FA), smoking (30% versus 19%) and alcohol consumption (8% versus 3%) (Table 2).

Regarding perinatal outcomes (Table 5), FA was associated with place of birth; FAs had more hospital deliveries than non-FAs (59% FA versus 52% non-FA) and fewer home deliveries (5% versus 12%). FA was also associated with medical pain relief (42% FA versus 26% non-FA), dilatation time (8 versus 6 h), transfer during labour (56% versus 49%), episiotomy (24% versus 16%), Apgar score ≤ 7 (5% versus 2%), and birth weight under the 10th percentile (6% versus 11%).

**Table 5:** Perinatal outcomes of frequent attenders and non-frequent split by referral (N = 1015)

	Non-referred frequent attender N = 107 (10.5%)	Non-referred non-frequent attender N = 431 (42.5%)	***P*** ***value***	Referred frequent attender N = 132 (13.0%)	Referred non-frequent attender N = 345 (34.0%)	***P value***
Apgar Score after 5 min*			.19			.27
< 8	5 (4.7)	10 (2.3)		2 (1.5)	12 (3.5)	
**Perinatal death**	1 (0.9)	4 (0.9)	1	0 (0.0)	6 (1.7)	.13
**Birthweight** (in percentiles)*			.13			.79
P < 10	6 (5.7)	45 (10.5)		13 (9.9)	37 (10.9)	
Mode of delivery *			.76			.34
Spontaneous	87 (82.1)	366 (85.1)		87 (65.9)	224 (65.1)	
Vaginal assisted birth	13 (12.3)	43 (10.0)		9 (6.8)	38 (11.0)	
Caesarean section	6 (5.7)	21 (4.9)		36 (27.3)	82 (23.8)	
Perineum*			.26			.03
Not ruptured	50 (49.0)	216 (52.9)		59 (60.8)	117 (44.2)	
Ruptured	28 (27.5)	125 (30.6)		20 (20.6)	75 (28.3)	
Episiotomy	24 (23.5)	67 (16.4)		18 (18.6)	73 (27.5)	
Haemorrhage*	5 (4.7)	22 (5.2)	.84	7 (5.4)	35 (10.4)	.10
**Place of birth**			.01			.34
Home	5 (4.7)	52 (12.1)		1 (0.8)	0 (0.0)	
Birth centre	37 (36.4)	156 (36.2)		7 (5.3)	14 (4.1)	
Hospital	63 (58.9)	223 (51.7)		124 (93.9)	331 (95.9)	
Transfer during labour*	60 (56.1)	210 (48.7)	.21	NA	NA	
Induction of labour*	NA	NA		96 (73.3)	205 (59.4)	.02
Medical pain relief*	45 (42.1)	112 (26.0)	.01	79 (60.3)	179 (52.0)	.40
**Gestational age at onset of labour in weeks** mean (sd)	38.9 (2.6)	39.3 (2.1)	.05	38.6 (1.9)	38.7 (2.8)	.67
**Duration of dilatation in hours** median (IQR)	8.0 (5.0–11.0)	6.0 (4.0–9.0)	<.01	6.0 (4.0–9.0)	6.0 (4.0–9.0)	.48
**Duration of expulsion in minutes** median (IQR)	17.0 (8.8–39.0)	15.0 (7.0–37.0)	.40	14.0 (7.0–32.0)	17.0 (8.0–33.0)	.18

*sample size varies due to missing data; valid percentages are shown

**SD = standard deviation; IQR = interquartile range

### Referred pregnant women

Among the referred group (*N* = 477), FA was associated with a Dutch background (56% FA versus 42% non-FA), living in a deprived area (17% versus 27%), psychosocial problems (25% versus 19%), a history of sexual violence (24% versus 12%), smoking (33% versus 22%), alcohol consumption (8% versus 3%), and drug addiction (6% versus 2%) (Table 2).

Regarding perinatal outcomes (Table 5), FA was significantly associated with induction of labour (73% FA versus 59% non-FA), episiotomy (19 and 28%) or ruptured perineum (21 and 28%), C-section (27 and 24%), vaginal assisted birth (7 and 11%), medical pain relief (60% versus 52%) and haemorrhage (5% versus 10%). FAs had less often a baby with an Apgar Score ≤ 7 (2% FA versus 4% non-FA), and perinatal death (0% versus 2%). Main reasons for transfer during labour were a request for pain relief (28%), insufficient progress of dilatation (25%) and meconium-stained fluid (14%).

### Regression analysis of estimation of FA on obstetric and neonatal outcomes

The results of the regression analyses, unadjusted and adjusted for medical and sociodemographic factors, are presented in Table 6. Mode of delivery was made binary due to the low prevalence of vaginal assisted birth which was therefore grouped with C-section as ‘assisted birth’. Duration of dilatation and expulsion were non-normally distributed and thus, log-transformed to comply with the linear model assumptions. No regression models were conducted for perinatal death and low Apgar score due to their low prevalence. Furthermore, no regression models were conducted for gestational age at onset of labour as there was minor variation between the groups of women and they were not clinically relevant. Only the adjusted effects that were significantly associated or of substantial size (more than half as likely) are discussed.

**Table 6:** Unadjusted and adjusted associations between frequent attendance/non-frequent attendance and perinatal outcomes, stratified by referral

	Non-referred		Referred	
	Unadjusted n ≤ 538*	Adjusted n ≤ 538*	Unadjusted n ≤ 477*	Adjusted n ≤ 477*
	OR (95%CI)	OR (95%CI)	OR (95%CI)	OR (95%CI)
Mode of delivery*				
Spontaneous				
Assisted birth^#^	1.25 (0.71–2.19)	1.14 (0.63–2.07)	0.97 (0.64–1.47)	0.94 (0.60–1.47)
**Birthweight** (in percentiles)*				
p ≥ 10				
* p* > 10	0.51 (0.21–1.24)	0.51 (0.21–1.24)	0.90 (0.46–1.76)	0.93 (0.47–1.84)
Perineum*				
Not ruptured				
Ruptured	0.97 (0.58–1.62)	1.05 (0.61–1.80)	0.53 (0.30–0.95)	0.50 (0.27–0.95)
Episiotomy	1.55 (0.89–2.71)	1.53 (0.80–2.94)	0.49 (0.27–0.89)	0.48 (0.24–0.95)
Haemorrhage*				
No				
Yes	0.91 (0.34–2.47)	0.97 (0.37–2.78)	0.50 (0.21–1.15)	0.52 (0.22–1.21)
**Place of birth** Home/birth centre				
Hospital	1.34 (0.87–2.05)	1.36 (0.87–2.12)	NA	NA
Transfer during labour*				
No				
Yes	1.34 (0.87–2.06)	1.31 (0.85–2.03)	NA	NA
Induction of labour*				
No				
Yes	NA	NA	1.87 (1.20–2.92)	1.86 (1.17–2.95)
Medical pain relief*				
No				
Yes	2.06 (1.34–3.20)	1.98 (1.24–3.17)	1.40 (0.93–2.11)	1.38 (0.88–2.15)
**Log duration of dilatations in hours**	1.20 (1.04–1.38)	1.20 (1.04–1.38)	1.03 (0.87–1.21)	1.03 (0.87–1.21)
**Log duration of expulsion in minutes**	1.06 (0.83–1.36)	1.06 (0.83–1.36)	0.91 (0.70–1.20)	0.91 (0.70–1.20)

*sample size varies due to missing data; ^#^vaginal assisted birth and Caesarean section together; OR = odds ratio, CI = confidence intervals 95%

No regression analysis for:

Apgar score

Perinatal death

Gestational age

In the non-referred group, FA compared to non-FA was significantly associated with medical pain relief (OR = 1.98 (1.24–3.17)) and longer dilatation time (OR = 1.20 (1.04–1.38)). There were no significant differences by health care utilisation in low birth weight, place of birth, transfer during labour, mode of delivery, haemorrhage, perineum, and duration of expulsion.

In the referred group, FA compared to non-FA was significantly associated with induction of labour (OR = 1.86 (1.17–2.95)), ruptured perineum (OR = 0.50 (0.27–0.95)) and episiotomy (OR = 0.48 (0.24–0.95)). FA was not significantly associated with haemorrhage, medical pain relief, mode of delivery, birth weight, and durations of dilatation and expulsion.

## Discussion

To our best knowledge, our study is the first to examine the associations between FA perinatal outcomes and FA in midwifery-led care. Nearly a quarter (24%) of the included women in our study were considered FAs and received more prenatal visits than the national recommendations. In the group of women who were not referred to an obstetrician during the pregnancy, FA was significantly associated with medical pain relief and duration of dilatation. In the referred group, FA was significantly associated with induction of labour, ruptured perineum and episiotomy.

### Strengths and limitations

Our study addressed the non-consistent results of the current literature on the effect of FA on perinatal outcomes in midwifery-led care. It utilised high-quality data from a medical database and had a negligible amount of missingness. Since the database included complete medical and background information, it was possible to accurately identify the number of consultations and to examine its relationship with perinatal outcomes. The cohort was described previously [23] and is representative for pregnant women seen in primary midwifery care in the Netherlands, except for the almost twice as high prevalence of non-Dutch women allowing to study differences by ethnicity [23, 41].

The main limitation of this exploratory study is the relatively small study population (*n* = 1015), that means that relatively rare outcomes such as suboptimal Apgar score and perintal death could not be assessed. As this study included only one midwifery practice, generalisation to all pregnant women should be made with caution. Another limitation is that the reasons for induction of labour and C-section were unknown.

### Comparison with literature

The literature lacks studies concerning non-referred and referred FAs in prenatal healthcare, making it difficult to directly compare our result with others. Our study showed that FA in the non-referred group was associated with medical pain relief. One possible explanation might be the higher rates of sexual violence among non-referred FAs compared to non-referred non-FAs (16% vs 9%) as sexual violence is associated with higher rates of general or lifetime anxiety [42, 43]; there is a significantly higher demand for pain relief among women who were anxious during pregnancy [44, 45]. Furthermore, non-referred FAs in our study had a significantly longer primary stage of labour, and pain relief might prolong delivery [46].

In the Dutch maternity care system, if a low-risk pregnant woman has a request for induction of labour, she will be referred to an obstetrician. In line with a large cohort study in obstetric care, FAs in the referred group in our study had more often an induction of labour [27]. The higher rates of induction could be explained by gestational stress, one of the underlying reasons for FA [23]. In this context, gestational stress means that the level of stress experienced by the pregnant woman is higher than she can cope with [47].

Over the last years, studies have examined the exposure to episiotomies in midwifery-led care and obstetric-led care settings and demonstrated that women in which labour was induced and who received regional analgesia have significantly higher rates of episiotomy [48, 49]. Our study showed higher rates of induced labour and demand for medical pain relief among referred FAs. However, contrary to earlier results, we found a significant lower episiotomy rate and ruptured perineum. The associations between the provision of pain relief, ruptured perineum and episiotomy need to be examined in general but also in FA.

Contrary to other research, we did not find an association between FA and C-section or vaginal assisted birth [27, 35]. The discrepancy may be explained by different factors. One important factor could be differences in cut-off points to categorise FA. Carter et al. used a cut-off point of more than 10 prenatal visits (> 10) to define FA, based on the median of the study population, instead of using the Kotelchuck-Index [50]. Moreover, the 10 prenatal visits were lower than the 12 prenatal visits recommended by the ACOG for women who started prenatal care in the first trimester [51]. Additionally, Carter et al. used a different control group than our study: all pregnant women with 10 or less prenatal visits (adequate and inadequate together), whereas our control group existed only of women with the adequate (and recommended) number of prenatal visits. Different interpretations of FA highlights the need for an internationally accepted and established definition of FA to be able to compare studies, establish relevant risk factors, and inform clinical guidelines.

### Future research

Our study was exploratory, and therefore it is recommended to repeat our study in a larger setting to improve the external validity of the results. Furthermore, this would allow to study relatively rare perinatal outcomes, e.g. suboptimal Apgar score and perinatal death, and result in more robust conclusions on the relationship between FA and obstetric and neonatal outcomes. We advise future studies to include the moment (gestational age) at which women are referred to an obstetrician in the analyses. Also, research about the underlying mechanisms of FA has to be performed, targeted explicitly on provider-related factors and client-related factors which could contribute to overuse of prenatal 

## Conclusion

Perinatal outcomes differed by perinatal healthcare utilisation and antenatal referral to an obstetrician. Non-referred FAs had relatively more often medical pain relief and longer duration of dilatation, whereas referred FAs had relatively more often an induction of labour. Further research within a larger study population is needed to assess possible associations between FA and rare adverse birth outcomes such as perinatal death and low Apgar score. FA influences the perinatal health care system; further research is needed on how professionals and management should organise prenatal care for FAs to improve care; research should also focus on the experiences and needs of FAs.

## Supplementary information

**Additional file 1.**

## Data Availability

The datasets used and analysed during the current study are available from the corresponding author on reasonable request.

## Abbreviations

FA: Frequent Attendance
Non-FA: Non-frequent Attendance
KNOV: Koninklijke Organisatie van Verloskundigen - the Royal Dutch Organisation of Midwives
KI-R: The Kotelchuck Index-Revised
