# Supplementary material for: Perinatal outcomes of frequent attendance in midwifery care in the Netherlands: a retrospective cohort study
Source: BMC Pregnancy Childbirth. 2020 May 6;20:269. doi: 10.1186/s12884-020-02957-1 (PMC7201569; doi:10.1186/s12884-020-02957-1)
Supplement: Supplementary file 1 — Additional file 1. [file 12884_2020_2957_MOESM1_ESM.docx]

**Appendix 1 Index for assessment of the adequacy of prenatal care utilization in the Dutch primary midwifery care context (Feijen-de Jong et al., 2015)**

| **Duration of gestation** | | **Initiation of care** | **Number of visits** | **Kotelchuck Index*^2^*** |
| --- | --- | --- | --- | --- |
| **0 - 11+6** | | ≤ 11+6 | ≥ 3 | 4 |
|  |  |  | 1-2 | 3 |
|  |  |  |  | 2 |
|  |  |  | 0 | 1 |
| **12 - 26+6** | | ≤ 11+6 | ≥ 6 | 4 |
| **Ideally 3.75 visits*^1^*** | |  | 3-5 | 3 |
|  | |  | 2 | 2 |
|  | |  | ≤ 1 | 1 |
|  | | ≥ 12+0 |  | 1 |
| **27 - 36+6** | |  | ≥ 10 | 4 |
| **Ideally 7.5 visits*^1^*** | |  | **6-9** | 3 |
|  | |  | 4-5 | 2 |
|  | |  | ≤ 3 | 1 |
|  | | ≥ 12+0 |  | 1 |
| **37+0 - 37+6**  **Ideally 11 visits*^1^*** | | ≤ 11+6  ≥ 12+0 | ≥ 13  10-12  6-9  ≤5 | 4  3  2  1  1 |
| **38+0 - 38+6**  **Ideally 12 visits*^1^*** | ≤ 11+6  ≥ 12+0 | ≥ 14  10-13  6-9  ≤5 | 4  3  2  1  1 |  |
| **39+0 - 39+6**  **Ideally 13 visits*^1^*** | ≤ 11+6  ≥ 12+0 | ≥ 15  11-14  7-10  ≤ 6 | 4  3  2  1  1 |  |
| **40+0 - 40+6**  **Ideally 14 visits*^1^*** | ≤ 11+6  ≥ 12+0 | ≥ 16  12-15  7-11  ≤ 6 | 4  3  2  1  1 |  |
| **41+0 - 41+6**  **Ideally 15 visits*^1^*** | ≤ 11+6  ≥ 12+0 | ≥ 17  **12-16***  **8-11**  ≤ 7 | 4  3  2  1  1 |  |

*^1^*According to the guidelines of the Royal Dutch Organization of Midwives
*^2^* Kotelchuck Index:

1. Inadequate (received less than 50% of expected visits)
2. Intermediate (50%-79%)
3. Adequate (80%-109%)
4. Adequate Plus (110% and more)
